# Efficacy and Safety of Sodium Glucose Cotransporter-2 (SGLT2) Inhibitors in Patients With Diabetes and Chronic Kidney Disease (CKD): A Meta-analysis of Randomized Control Trials

**DOI:** 10.7759/cureus.31898

**Published:** 2022-11-26

**Authors:** Sidra Jamil, Arfa Zainab, Avneet Kaur Manjeet Singh Arora, Tanveer Ahamad Shaik, Vimal Khemani, Favour C Mekowulu, Yared N Aschalew, Saima Khan

**Affiliations:** 1 Internal Medicine, California Institute of Behavioral Neurosciences & Psychology, Fairfield, USA; 2 Medicine, Mohammad ud din Islamic Medical College, Mir Pur, PAK; 3 Public Health, Epidemiology, University of California Berkeley, Berkeley, USA; 4 Internal Medicine, Mahatma Gandhi Mission's Medical College, Navi Mumbai, IND; 5 Cardiovascular Medicine, University of Louisville School of Medicine, Louisville, USA; 6 Internal Medicine, Jinnah Sindh Medical University, Karachi, PAK; 7 Internal Medicine, V.N. Karazin Kharkiv National University, Kharkiv, UKR; 8 Internal Medicine, Inova Alexandria Hospital, Alexandria, USA; 9 Internal Medicine, Sir Syed College of Medical Sciences for Girls, Waterbury, USA

**Keywords:** safety, efficacy, chronic kidney disease, diabetes, sodium-glucose cotransporter-2 (sglt2) inhibitors

## Abstract

The current meta-analysis aims to assess the efficacy and safety of sodium glucose cotransporter 2 (SGLT2) inhibitors in individuals with diabetes and chronic kidney disease (CKD). The current meta-analysis was conducted using the Preferred Reporting Items for Systematic Reviews and Meta-Analyses (PRISMA) guidelines. A systematic search was conducted to identify all relevant studies related to the efficacy and safety of SGLT2 inhibitors in individuals with diabetes and CKD. The search was undertaken in PubMed, EMBASE, and Cochrane Library from January 2000 to September 2022. The primary efficacy outcome assessed in the current meta-analysis included major adverse cardiovascular events (MACE). Other efficacy outcomes included all-cause mortality and change in hemoglobin A1c (HbA1c) (%). Safety outcomes included serious adverse events, acute kidney injury, hypoglycemia, and hyperkalemia. In total 11 articles met the inclusion criteria and were included in the final analysis enrolling 27520 patients (14491 in the SGLT2 inhibitors and 13029 in the placebo group). The findings of this meta-analysis have shown that the risk of MACE and all-cause mortality was significantly lower in patients receiving SGLT2 inhibitors. Additionally, Hb1AC change was also significantly greater in SGLT2 inhibitors group. In relation to safety outcomes, serious adverse events, risk of acute kidney injury, and hyperkalemia were significantly lower in the SGLT2 inhibitors group. The SGLT2 inhibitors significantly decreased the risk of major cardiovascular events and all-cause mortality in patients with CKD and diabetes. Furthermore, SGLT2 inhibitor is also effective in reducing Hb1Ac levels in patients.

## Introduction and background

Diabetes is a major risk factor for microvascular and macrovascular diseases [1]. Diabetes is a metabolic illness that affects more than 400 million individuals around the world [2]. It is estimated that by 2045, its prevalence will be increased by 700 million people [3]. Chronic kidney disease (CKD), which is defined as the sustained loss of kidney function over a long period of time or the presence of albuminuria or other indications of renal damage, affects 16% of the general population [4]. Diabetes is one of the common causes of CKD and it accounts for up to 50% of individuals who develop end-stage renal disease [5]. Individuals with CKD and diabetes are at higher risk of cardiovascular outcomes including heart failure, atherosclerotic cardiovascular disease, and mortality [6].

Sodium-glucose cotransporter 2 (SGLT2) inhibitors reduce hyperglycemia in patients with diabetes. It acts by reducing the renal reabsorption of glucose, thus increasing the urinary excretion of glucose [7]. The use of SGLT2 inhibitors is associated with a lowering of glycated hemoglobin in patients with diabetes including individuals with stage 2 and stage 3 CKD [8]. Different SGLT2 inhibitors have been approved by the European Medicines Agency (EMA) and the Food and Drug Administration (FDA) for the management of glycemic control in patients with type 2 diabetes [9]. In order to prevent renal and cardiovascular outcomes in persons with type 2 diabetes and CKD who have not attained glycemic objectives, the American Diabetes Association supports the use of SGLT2 inhibitors along with metformin, which is also in accordance with the KDIGO 2022 draught recommendations [10].

Recent trials have shown a lower risk of major cardiovascular events with oral antihyperglycemic agents. Studies assessing SGLT2 inhibitors reported a decreased risk of the primary composite endpoint of fatal and non-fatal myocardial infarction, stroke, or cardiovascular deaths compared to placebo when added to standard antihyperglycemic treatment in patients with type 2 diabetes [11-12].

To date, limited evidence is there related to the safety and efficacy of SGLT2 inhibitors in individuals with CKD and diabetes. The effect of SGLT2 inhibitors in individuals with CKD is different compared to other higher-risk individuals because of the enhanced prevalence of proteinuria, oxidative stress, inflammation, and mineral metabolism abnormalities. Recent studies have been conducted in this setting [13-17], posing an equal balance between the advantages and disadvantages of SGLT2 inhibitors in patients with diabetes and CKD, to review the certainty of the updated evidence in this setting for the benefit of stakeholders, such as patients, healthcare professionals, and policy-makers. This meta-analysis aims to assess the efficacy and safety of SGLT2 inhibitors in individuals with diabetes and CKD.

## Review

Methodology

The current meta-analysis was conducted using the Preferred Reporting Items for Systematic Reviews and Meta-Analyses (PRISMA) guidelines.

Search strategy

A systematic search was conducted to identify all relevant studies related to the efficacy and safety of SGLT2 inhibitors in individuals with diabetes and CKD. The search was undertaken in PubMed, EMBASE, and Cochrane Library from January 2000 to September 2022. Key terms used to carry out the search included “SGLT2 inhibitors,” “glucose,” “diabetes,” “chronic kidney disease,” and “cardiovascular outcomes.” The reference lists of all the included studies were also manually searched for possible inclusion.

Study selection

Two authors independently carried out the search. After identifying all relevant articles, titles and abstracts were screened for inclusion and exclusion criteria. Full texts of all eligible articles were retrieved to determine whether they are eligible to be included in the current meta-analysis or not. Articles included in the current meta-analysis if they fulfilled the following inclusion criteria: a) RCT studies assessing the efficacy and safety of SGLT2 inhibitors in patients with CKD and type 2 diabetes and b) Studies with at least six months follow-up period. Observational studies, quasi-experimental studies, and case reports were excluded from the current meta-analysis. Studies that did not report the desired outcomes (major cardiovascular outcomes, all-cause mortality, change in Hb1AC level, and safety events) were not included in the current meta-analysis. CKD is defined as an estimated glomerular filtration rate (eGFR) of less than 60 mL/min/1.73 m2 or urine albumin-to-creatinine ratio (UACR) of more than 30 mg/g.

Risk of bias assessment

The risk of bias was assessed by two authors independently using the Cochrane Collaboration tool for assessing the risk of bias. Domains assessed included sequence generation, allocation concealment, blinding of outcome assessor, blinding of patients, incomplete outcome data, selective outcome reporting, and other biases. They were graded as high, low, and unclear risk of bias. Any disagreement between the two authors was resolved through discussion.

Outcomes

The primary efficacy outcome assessed in the current meta-analysis included major adverse cardiovascular events (MACE). MACE is defined as a composite of cardiovascular death, nonfatal stroke, and nonfatal myocardial infarction. Other efficacy outcomes included all-cause mortality and change in HbA1c (%). Safety outcomes included serious adverse events, acute kidney injury, hypoglycemia, and hyperkalemia.

Data extraction

Data extraction was done by one author using pre-designed forms created on Microsoft Excel. Filled forms were checked by a second author. Data extracted from the eligible articles included: author name, year of publication, groups, sample size, follow-up period, characteristics of patients, and outcome variables.

Data analysis

Data analysis was performed using RevMan (version 5.4.0, the Cochrane Collaboration, London, United Kingdom). For binary efficacy outcomes, we sought to identify hazard ratios (HR) with their 95% confidence interval (95% CI) of the impacts of SGLT2 inhibitors in patients with CKD and diabetes from individual studies. Pooled treatment effects for safety endpoints were compared using risk ratios (RRs) and their 95% CIs. Pooled treatment effects for continuous outcomes were estimated using mean difference and their 95% CI. The pooled effect estimates and associated 95% CI were computed using random-effects meta-analysis models. For each outcome, a p-value < 0.05 was considered statistically significant. Heterogeneity was assessed using I-square statistics. I-square values of 0-25%, 25%-75%, and >75% were regarded as low, moderate, or high heterogeneity among the study results. Cochran-Q statistics was used for testing heterogeneity among the study results. A p-value < 0.1 was considered significant for heterogeneity among the study results.

Results

**Figure 1:**
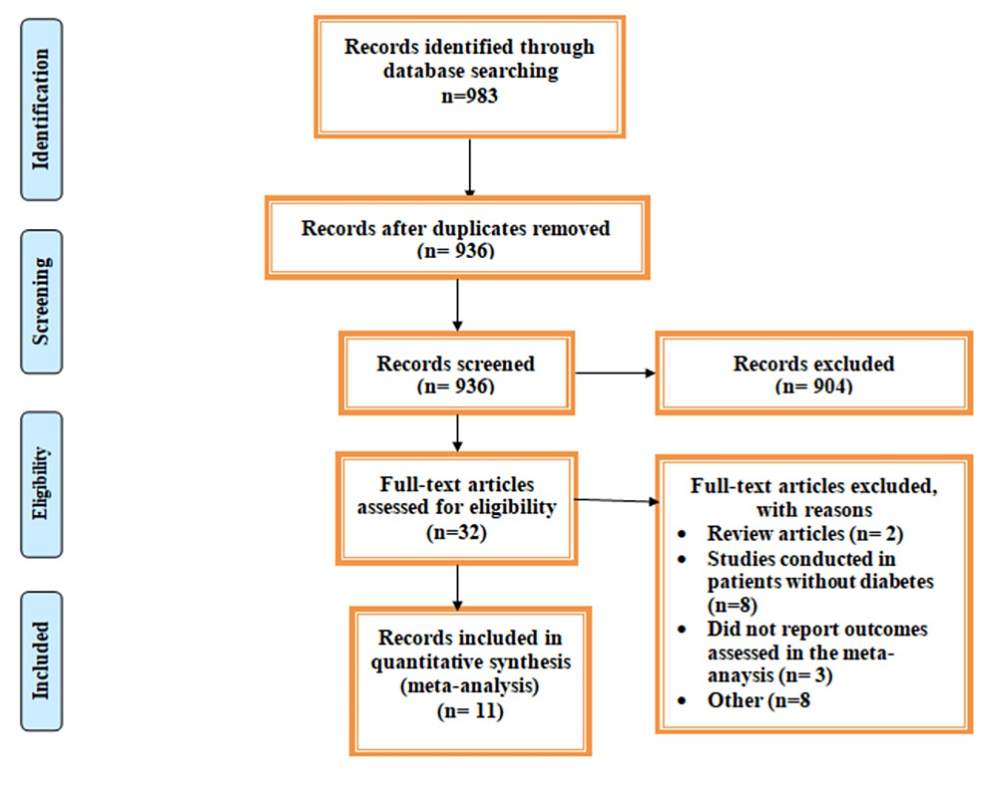
PRISMA flowchart of selection of studies. PRISMA, Preferred Reporting Items for Systematic Reviews and Meta-Analyses

Figure 1 shows PRISMA flowchart of selection of studies. A total of 983 articles were retrieved from online searching. After removing duplicates, title and abstract screening of 936 articles were done to assess the eligibility criteria. Thirty-two articles were included for full-text screening. A total of 11 articles met the inclusion criteria and were included in the final analysis enrolling 27520 patients (14491 in the SGLT2 inhibitors and 13029 in the placebo group) [12-22]. We included one study twice in the analysis, as the analysis was done separately on patients with stage 2 and stage 3 CKD [13]. 

**Figure 2:**
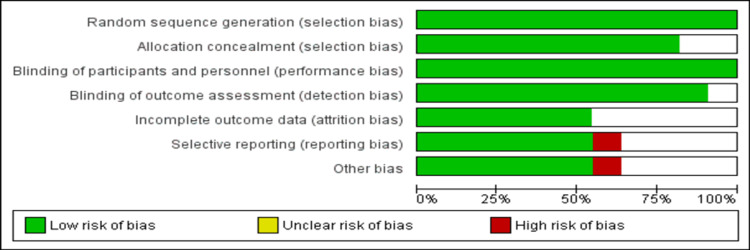
Assessment of bias.

We included two studies of empagliflozin, three studies of dapagliflozin, three studies of canagliflozin, and one study each of sotagliflozin, ertugliflozin, and luseogliflozin. Table 1 shows the characteristics of all included studies. The pooled mean age of included patients was 65.81 years. A majority of patients in all the included studies were males. Among the nine included trials, follow-up ranged from six months to 48 months. Figure 2 shows the risk of bias of all included studies. Overall the risk of bias was low in the current meta-analysis.

**Table 1:** Characteristics of included studies. SGLT2, sodium glucose cotransporter 2; CKD, chronic kidney disease

Author name	Year of publication	Setting	Stage of CKD	Groups	SGLT2 type	Sample size	Follow-up	Mean age (in years)	Males n (%)
Barnett et al. (a) [13]	2014	Multicenter	Stage 2	SGLT2	Empagliflozin	97	12 months	62	117 (60.9)
				Placebo		95			
Barnett et al. (b) [13]	2014	Multicenter	Stage 3	SGLT2	Empagliflozin	187	12 months	64.8	213 (57.0)
				Placebo		187			
Bhatt et al. [14]	2020	Multicenter	Stage 3	SGLT2	Sotagliflozin	5292	16 months	69	5830 (55.1)
				Placebo		5292			
Cannon et al. [15]	2020	Multicenter	Stage 3	SGLT2	Ertugliflozin	1199	42 months	68.1	1142 (64.3)
				Placebo		608			
Fioretto et al. [16]	2018	Single Center	Stage 3	SGLT2	Dapagliflozin	160	6 months	65.8	182 (56.7)
				Placebo		161			
Haneda et al. [17]	2016	Single Center	Stage 3	SGLT2	Luseogliflozin	95	6 months	68	111 (76.6)
				Placebo		50			
Heerspink et al. [18]	2020	Multicenter	Stage 2 and Stage 3	SGLT2	Dapagliflozin	2152	29 months	61.8	2879 (66.9)
				Placebo		2152			
Neal et al. [12]	2017	Multicenter	Stage 3	SGLT2	Canagliflozin	1110	48 months	NR	NR
				Placebo		929			
Perkovic et al. [19]	2019	Multicenter	Stage 3	SGLT2	Canagliflozin	2202	32 months	63	2907 (64.1)
				Placebo		2199			
Wanner et al. [20]	2016	Multicenter	Stage 3	SGLT2	Empagliflozin	1212	37 months	67.1	1234 (67.8)
				Placebo		607			
Wiviott et al. [21]	2019	Multicenter	Stage 3	SGLT2	Dapagliflozin	606	12 months	NR	NR
				Placebo		659			
Yale et al. [22]	2013	Multicenter	Stage 3	SGLT2	Canagliflozin	179	6 months	68.5	163 (60.6)
				Placebo		90			

**Figure 3:**
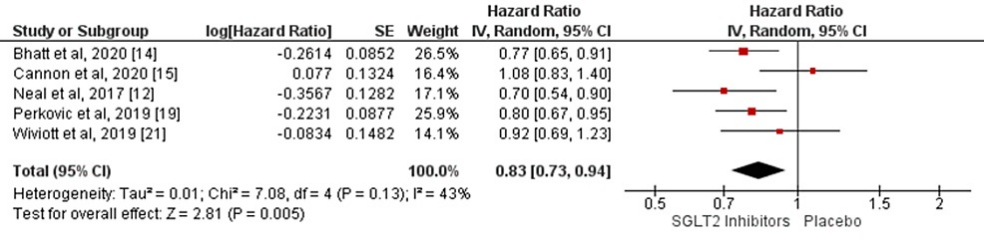
Forest plot showing the effect of SGLT2 inhibitors on MACE. SGLT2, sodium glucose cotransporter 2; MACE, major adverse cardiovascular event Sources: References [12, 14-15, 19, 21]

Five of the included studies compared the hazard of MACE between SGLT2 inhibitors and placebo [12, 14-15, 19, 21]. Analysis of associations between SGLT2 inhibitors and outcomes on the hazard for MACE is shown in Figure 3. Overall, the hazard of MACE was significantly lower in patients receiving SGLT2 inhibitors compared to the placebo group (HR: 0.83, 95% CI: 0.73-0.94, p-value = 0.005). The heterogeneity was moderate among the study results (I-square: 43%). Cochran Q-statistics did not show any significant heterogeneity among the study results (p-value = 0.13).

**Figure 4:**
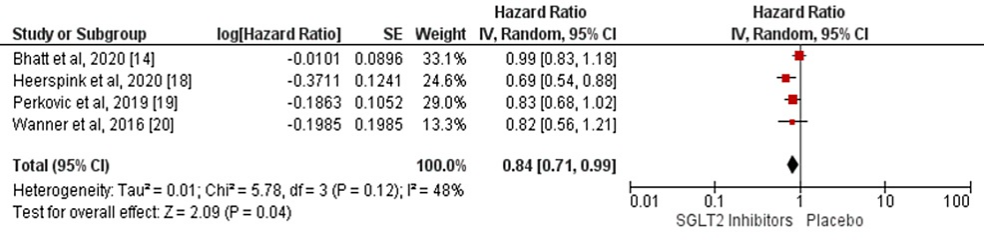
Forest plot showing the effect of SGLT2 inhibitors on all-cause mortality. SGLT2, sodium glucose cotransporter 2 Sources: References [14, 18-20]

Four studies compared the hazard of all-cause mortality between SGLT2 inhibitors and placebo [14, 18-20]. Analysis of associations between SGLT2 inhibitors and all-cause mortality is shown in Figure 4. Overall, the hazard of MACE was significantly lower in patients receiving SGLT2 inhibitors compared to the placebo group (RR: 0.84, 95% CI: 0.71-0.99, p-value = 0.04). The heterogeneity was moderate among the study results (I-square: 48%). No significant heterogeneity was reported among the study results (p-value = 0.12).

**Figure 5:**
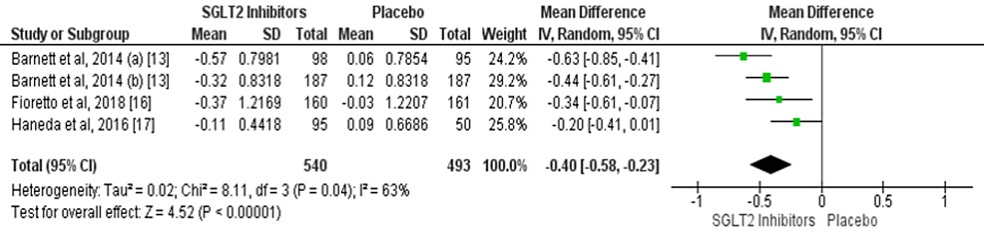
Forest plot on association of SGLT2 inhibitors on reduction of HbA1C. SGLT2, sodium glucose cotransporter 2; HbA1C, hemoglobin A1C Sources: References [13, 16-17]

Data on mean change in HbA1c were reported by three studies [13, 16-17]. The mean reduction of Hb1AC was significantly greater in patients receiving SGLT2 inhibitors compared to the patients receiving placebo (mean difference: -0.40, 95% CI: -0.58, -0.23, p-value: 0.001) as shown in Figure 5. The heterogeneity was moderate among the study results (I-square = 63%). Significant heterogeneity was found among the study results (p-value = 0.04).

Association of SGLT2 inhibitors with safety outcomes 

**Figure 6:**
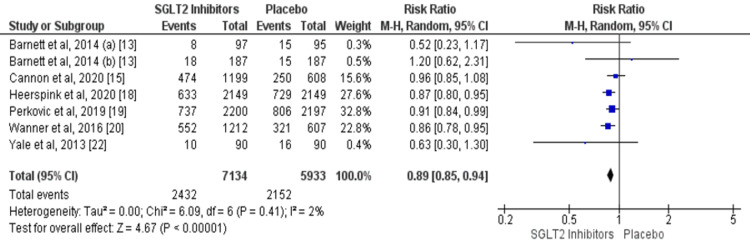
Comparison of serious adverse events between SGLT2 inhibitors and placebo group. SGLT2, sodium glucose cotransporter 2 Sources: References [13, 15, 18-20, 22]

A total of six trials compared data on serious adverse events among participants with diabetes and CKD [13, 15, 18-20, 22]. A lower risk of serious adverse events was reported in patients receiving SGLT2 inhibitors compared to the placebo group (RR: 0.89, 95% CI: 0.85-0.94, p-value = 0.001) as shown in Figure 6. Heterogeneity was low among the study results (I-square = 2%). No significant heterogeneity was reported among the study results (p-value = 0.41). Table 2 showed the effect of SGLT2 inhibitors on the risk of acute kidney injury, hypoglycemia, and hyperkalemia. The risk of acute kidney injury and hyperkalemia was significantly lower in patients in the SGLT2 inhibitors group.

**Table 2:** Effect of SGLT2 inhibitors on adverse events. RR, risk ratio; CI, confidence interval; SGLT2, sodium glucose co-transporter 2 *significant difference at p-value < 0.05

Events	Number of studies	RR	95% CI	p-value	I-square
Acute kidney injury*	3	0.81	0.67-0.98	0.031	0%
Hypoglycemia	5	0.91	0.82-1.02	0.101	35%
Hyperkalemia*	3	0.77	0.63-0.93	0.008	27%

Discussion

The current meta-analysis includes the comparison of clinical outcomes data of SGLT2 inhibitors and placebo among patients with diabetes and CKD. Compared to a placebo, SGLT2 inhibitors probably reduce HbA1c, risk of MACE, and all-cause mortality. This is largely consistent with past systematic reviews of the safety and efficacy of SGLT2 inhibitors including patients with diabetes conducted by Shyangdan et al. [23] and Storgaard et al. [24]. Similarly, a recent meta-analysis of four studies found that SGLT2 inhibitors are effective to protect individuals with type 2 diabetes and CKD from MACE [25].

The present study results augment the rising evidence base that in general, SGLT2 inhibitors are associated with favorable cardiovascular outcomes in patients with diabetes and CKD. Options for anti-diabetes treatment in individuals with CKD are limited and adjustment of dose is required for several oral drugs [26]. Multimodal treatment such as the optimization of lipids and blood pressure is required in individuals at high cardiovascular risk [27].

Management of CKD in primary care needs to include reduction of cardiovascular risk, management of hypertension, diabetes and many other comorbidities; circumventing nephrotoxins, making sure of the correct dosage of medicines; and monitoring kidney functions and other relevant laboratory tests [25]. Based on recent evidence, as noted in the current meta-analysis, SGLT2 inhibitors are standard therapy for decreasing the hazard of adverse clinical outcomes from CKD [28].

Individuals with diabetes are at high risk of cardiovascular disease and heart failure compared with individuals without type 2 diabetes [29]. Out of five trials that assessed the impact of SGLT2 inhibitors in the current meta-analysis; three studies supported that SGLT2 inhibitors can reduce the risk of cardiovascular events [12, 14, 19]. The mechanism behind the beneficial impacts of SGLT2 inhibitors on MACE risk observed in the current meta-analysis is not clear but possibly due to the prevention of atrial fibrillation flutter (AFL) and atrial fibrillation (AF) [30].

The exact mechanisms for the beneficial effect of SGLT2 inhibitors on stroke risk observed in our study are unclear but may be mediated by the prevention of AF and AFL [31]. In addition, several hypotheses have been proposed as mechanisms for the improvement of cardiovascular outcomes by treatment with SGLT2 inhibitors [32-33]. For instance, osmotic diuresis because of glucosuria is likely to play a significant part in decreasing cardiac preload and thus decreasing the incidence of arrhythmia and heart failure, resulting in a reduction in cardiovascular outcomes [32]. A study reported that dapagliflozin 10 mg therapy decreased plasma volume by 9% [34]. An increase in hematocrit and blood ketone bodies may also contribute to cardioprotection by treatment with SGLT2 inhibitors [35].

With respect to safety outcomes, our meta-analysis has identified that the risk of serious adverse events was lower in SGLT2 inhibitors. In the SGLT2, rates of adverse events suggestive of acute kidney injury or hyperkalemia were lower than or comparable to those in the placebo group. Previous observational studies have also suggested that SGLT2 inhibitors are associated with a reduced risk of acute kidney injury [36-37]. Although the precise processes underlying this association are unknown, some ideas include a reduction in tubular injury and a milder ischemic-reperfusion injury to the kidney [36].

Society clinical guidelines have shown the kidney benefits of SGLT2 inhibitors in diabetic patients and to a lesser extent, in those individuals without diabetes [15]. The American Diabetes Association recommends the utilization of SGLT-2 inhibitors in stage 3 CKD patients and type 2 diabetes irrespective of glycemic control, for slowing the progression of CKD and decreasing the risk of heart failure [16].

The current meta-analysis has certain limitations. Firstly, most trials included in the current meta-analysis excluded patients with eGFR less than 30 mL/min/1.73 m2, thus we were not able to assess the effect of SGLT2 inhibitors on patients with stage 4 CKD. Secondly, the majority of the data in the current meta-analysis came from a subgroup analysis of large RCTs. This may affect the credibility of the results of the current study. Lastly, the limited number of studies for each outcome has limited our ability to do evocative subgroup analysis including analysis by gender, age, or comorbidities.

## Conclusions

The SGLT2 inhibitors significantly decreased the risk of major cardiovascular events and all-cause mortality in patients with CKD and diabetes. Furthermore, SGLT2 inhibitor is also effective in reducing Hb1AC levels in patients. For safety outcomes, SGLT2 inhibitors can significantly reduce the number of serious adverse events compared to patients receiving a placebo. The risk of acute kidney injury and hyperkalemia was significantly lower in patients receiving SGLT2. According to the findings of the current meta-analysis, SGLT2 inhibitors are the latest addition to the therapy used for the management of patients with CKD and diabetes.
